# Robot-assisted versus laparoscopic living donor nephrectomy: superior outcomes after completion of the learning curve

**DOI:** 10.1007/s11701-023-01681-0

**Published:** 2023-08-02

**Authors:** Elias Khajeh, Rajan Nikbakhsh, Ali Ramouz, Ali Majlesara, Mohammad Golriz, Beat P. Müller-Stich, Felix Nickel, Christian Morath, Martin Zeier, Arianeb Mehrabi

**Affiliations:** 1 Head of the Division for Abdominal Transplantation, Department of General, Visceral and Transplant Surgery, University Hospital Heidelberg, Im Neuenheimer Feld 420, 69120 Heidelberg, Germany; 2 Department of Nephrology, University of Heidelberg, Heidelberg, Germany

**Keywords:** Nephrectomy, Living donors, Learning curve, Minimally invasive surgical procedures, Robotic surgical procedures

## Abstract

The use of robots in donor nephrectomy has increased in recent years. However, whether robot-assisted methods have better outcomes than traditional laparoscopic methods and how surgical experience influences these outcomes remains unclear. This meta-analysis compares the outcomes of robot-assisted donor nephrectomy (RADN) with those of laparoscopic donor nephrectomy (LDN) and to investigate the effects of surgical experience on these outcomes. A systematic literature search was conducted in Medline (through PubMed) and Web of Science databases. Perioperative data were extracted for meta-analysis. To assess the impact of the learning curve, a subgroup analysis was performed to compare outcomes between inexperienced and experienced surgeons. Seventeen studies with 6970 donors were included. Blood loss was lower (mean difference [MD] = – 13.28, p < 0.01) and the warm ischemia time was shorter (MD = – 0.13, p < 0.05) in the LDN group than the RADN group. There were no significant differences in terms of conversion to open surgery, operation time, surgical complications, hospital stay, costs, and delayed graft function between the groups. Subgroup analysis revealed that operation time (MD = – 1.09, p < 0.01) and length of hospital stay (MD = – 1.54, p < 0.05) were shorter and the rate of conversion to open surgery (odds ratios [OR] = 0.14, p < 0.0001) and overall surgical complications (OR = 0.23, p < 0.05) were lower in experienced RADN surgeons than in experienced LDN surgeons. Surgical experience enhances the perioperative outcomes following RADN more than it does following LDN. This suggests that RADN could be the method of choice for living donor nephrectomy as soon as surgeons gain sufficient experience in robotic surgery.

**Supplementary Information:**

The online version contains supplementary material available at 10.1007/s11701-023-01681-0.

## Introduction

Kidney transplantation from either a deceased or a living donor is the treatment of choice in patients with end-stage renal disease [1]. Kidney transplant from a living donor has several advantages over transplantation from a dead donor, including higher patient/graft survival, lower rejection rates, and shorter warm ischemia time [2, 3]. However, it is important to maintain the health and safety of living donors so they can resume normal activities as soon as possible [4].

Many living donors undergo open donor nephrectomy; however, this method is associated with increased postoperative pain, scarring, and other adverse effects [5]. This limits the use of open donor nephrectomy because maintaining the health and safety of donors is crucial [4]. Minimally invasive techniques including laparoscopic donor nephrectomy (LDN) and robot-assisted donor nephrectomy (RADN) have improved the outcomes of living donation, including better cosmetic results, less intraoperative blood loss, reduced postoperative pain, and better patient recovery [6, 7].

Systematic reviews and meta-analyses have reported that RADN is associated with less postoperative pain but a longer operative time, longer warm ischemia time, and higher blood loss than LDN [5, 8, 9]. In addition, some studies have suggested that these inferior intraoperative outcomes of RADN, such as longer operative and warm ischemia time, could be due to the surgical learning curve, and that these disadvantages disappear as surgical experience increases [10, 11]. In contrast, other studies have reported that the surgical learning curve has no effect on RADN and LDN outcomes [12, 13]. To address these controversial findings, we designed a meta-analysis to compare the surgical outcomes of RADN and LDN and to evaluate the role of surgical experience on these outcomes.

## Materials and methods

The study protocol of the current systematic review was prospectively registered at PROSPERO (registration number: CRD42022376549) and this meta-analysis designed according to Preferred Reporting Items for Systematic Reviews and Meta-Analysis (PRISMA) 2020 guidelines and recommendations of the Study Center of the German Society of Surgery [14, 15].

First, a two-arm meta-analysis was carried out to compare the surgical outcomes of LDN and RADN. Then, a subgroup analysis was conducted to compare the outcomes in the following four subgroups: inexperienced LDN surgeons, experienced LDN surgeons, inexperienced RADN surgeons, and experienced RADN surgeons. We compared surgical outcomes between these subgroups to evaluate the effect of the learning curve and surgical experience on the meta-analysis results.

### Eligibility criteria

The study question was developed using the PICOS strategy (population, intervention, comparison, outcome, and study design).

The following criteria were used to determine whether a study should be included in the analysis:

- *Population:* living kidney donors who underwent minimally invasive donor nephrectomy
- *Intervention:* RADN
- *Comparator:* LDN
- *Outcome:* Perioperative outcomes in donors and delayed graft function in recipients
- *Study design:* All types of study, except case reports, editorials, and letters to the editor.

The studies were rigorously examined and duplicate publications and overlapping reports were excluded to ensure the same patients were not included twice.

### Literature search

The following search terms were used to perform a systematic literature search in Medline (through PubMed) and Web of Science: (((Kidney OR Renal) AND (Transplant* OR explant* OR harvest*)) OR ("donor nephrectomy")) AND ("robot" OR "robotic" OR "Da vinci" OR davinci). The search was not limited to a particular study category or publication year. The last search was made in April 2022.

### Study selection and data extraction

The primary electronic search was done by two investigators (RN and AR) using predefined keywords. The titles and abstracts of the extracted articles were reviewed according to the inclusion and exclusion criteria to identify relevant articles. Two authors (RN and AM) then screened the complete text of relevant articles and extracted the data. Discrepancies between these two investigators were resolved through discussions with the first author (EK). Study characteristics, patient characteristics, study quality, and the abovementioned surgical outcomes were all extracted for each study.

### Characteristic data

*Demographic characteristics*: Year of study, country of study, number of patients in each group, age, gender, preoperative body mass index (BMI) (kg/m^2^), right-side or left-side kidney explanation in donors, number of arteries, and follow-up time were extracted.

*Outcomes and data items:* Warm ischemia time (minutes), operation time (minutes) (defined as the time from initiation of surgery after laparoscopic setup to the end of surgery), estimated blood loss (ml), conversion rate to open surgery, length of hospital stay, overall complications (including intraoperative and postoperative complications), major complications (postoperative complications with a Clavien–Dindo grade ≥ 3), healthcare costs, and delayed graft function.

### Quality assessment and risk of bias

Study quality was assessed using the Cochrane tool for bias assessment in randomized studies and the Risk Of Bias in Non-Randomized Studies—of Interventions (ROBINS-I) tool in non-randomized studies by two investigators (EK and RN) [16]. If the study had a low risk of bias in all categories, the overall risk of bias was considered low. If there was potential bias in at least one domain, the study was considered to have some concerns of bias and if the study exhibited a high risk of bias in at least one domain or some concerns in many domains, it was assumed to have a high risk of bias. The quality of evidence for each outcome was evaluated by Grading of Recommendations, Assessment, Development and Evaluations (GRADE) (Supplementary Table 1).

### Statistical analysis

Statistical analysis was carried out using R software version 4.2.1 (R Foundation for Statistical Computing, Vienna, Austria). For dichotomous data, the effect sizes were reported as odds ratios (ORs) and for continuous data, as mean differences (MDs). Summary effect measures were reported with confidence intervals of 95%. For this meta-analysis, we calculated proportions using a random-effects model. Statistical heterogeneity was evaluated using χ^2^ and inconsistency analyses, and heterogeneity was considered significant if *p* < 0.05 and I^2^ value was larger than 50%. For subgroup analysis, we carried out a random-effects model of frequentist network meta-analysis to compare the four subgroups categorized according to surgical experience. Publication bias was evaluated using funnel plots. In all analyses, *p* values < 0.05 were considered significant.

## Results

### Characteristics of included studies

We screened 1408 articles in our systematic search. After duplicates were removed, 1082 articles were included in the title and abstract review. Of these, 203 articles were reviewed in-depth and 17 of these were included in the final meta-analysis (Supplementary Fig. 1). Table 1 summarizes the baseline characteristics of these 17 studies, which comprised 6,970 donors. In the subgroup analysis, six of the 17 included studies reported the effect of the learning curve on the outcomes of LDN and RADN. Most of these studies considered 20–25 operations as the cut-off number for reaching expertise in robotic surgery. Supplementary Table 2 summarizes the baseline characteristics of the six studies included in the subgroup analysis.

**Table 1:** Characteristics of included studies in the current meta-analysis

Study (year)	Study design	Country	Group	Age (mean ± SD)	Sex (M/F)	BMI (mean ± SD)	Right/ left kidney	Multiple arteries	Follow-up (Months)
Horgan et al. (2002) [30]	Retrospective	USA	LDN: 23	34 (22–51)	13/10	24.6 ± 3.5	NA	NA	NA
			RADN: 12	33 (21–55)	7/5	25.4 ± 5.6	NA	NA	
Geffner et al. (2011) [31]	Retrospective	USA	LDN: 35	43.6 ± 11.2	NA	27.3 ± 4.5	NA	NA	9
			RADN: 35	44.5 ± 11.7	14/21	28.4 ± 4.5	8/35	NA	
Liu, X. S. et al. (2012) [32]	Retrospective	USA	LDN: 20	40.7	NA	25.3	NA	1/20	60
			RADN: 5	34.8	NA	31.2	NA	0/5	
Monn et al. (2014) [33]	Retrospective	USA	LDN: 4021	40.8 ± 12	1553/ 2468	NA	NA	NA	NA
			RADN: 142	44 ± 13	71/71	NA	NA	NA	
Bhattu et al. (2015) [28]	RCT	India	LDN: 30	45.33 ± 9.37	7/23	27.62 ± 3.53	18/12	NA	9
			RADN: 15	46.47 ± 11.21	2/13	28.97 ± 5.16	9/6	NA	
Cohen et al. (2015)[34]	Retrospective	USA	LDN: 20	NA	NA	NA	3/17	3/20	48
			RADN: 100	NA	NA	NA	21/79	18/100	
Janki et al. (2017) [35]	Retrospective	Netherlands	LDN: 61	49.3 (22.0–72.0)	27/34	24.8 (18.5–35.0)	0/61	2/61	3
			RADN: 59	53.0 (19.0–76.0)	23/36	23.6 (17.9–29.4)	0/59	12/59	
P. Luke et al. (2018) [36]	RCT	Canada	LDN: 25	50 (26–68)	7/18	27.1 ± 3.8	5/20	4/25	24
			RADN: 14	51(41–64)	9/5	25.8 ± 3.4	0/14	3/14	
Yang et al. (2018) [29]	Retrospective	USA	LDN: 73	39.4 ± 11.3	44/29	27.5 ± 4	15/58	NA	12
			RADN: 22	38.2 ± 11.4	12/10	25.8 ± 4.4	2/20	NA	
Shin et al. (2019) [37]	Retrospective	South Korea	LDN: 45	42.5 (29.0–51.0)	33/12	26.2 (23.8–28.0)	4/41	NA	6–12
			RADN: 56	45.4 (31.0–53.0)	38/18	25.4 (24.2–27.7)	11/45	12/56	
Achit et al. (2020) [38]	RCT	France	LDN: 65	51.2	23/42	25.3	NA	20/65	NA
			RADN: 69	49.1	32/37	25.1	NA	21/69	
Zeuschner et al. (2021) [12]	Retrospective	Germany	LDN: 205	51 (21–78)	75/130	25.9 (17.6–36.1)	45/160	38/205	48
			RADN: 52	54 (20–70)	16/36	25.4 (17.6–36.7)	11/41	6/52	
Dumlu et al. (2021) [10]	RCT	Turkey	LDN: 20	44 (21–69)	9/11	25.8 ± 4.0	NA	3/20	NA
			RADN: 40	40 (24–72)	17/23	27.85 ± 4.3	NA	4/40	
Takagi et al. (2021) [39]	Retrospective	Netherlands	LDN: 1365	51.8 (41.1–61.2)	606/759	25.3 (23.1–28)	619/746	258/1365	NA
			RADN: 103	54.0 (40.3–62.4)	42/61	23.9 (22–26.3)	20/83	26/103	
Lecoanet et al. (2022) [11]	RCT	France	LDN: 1365	51.2	23/42	25.3	NA	20/65	NA
			RADN: 103	49.1	32/37	25.1	NA	21/69	
Thai et al. (2022)[40]	Prospective	Vietnam	LDN: 31	47.6 ± 10.1	13/18	23.2 ± 2.23	3/28	6/31	48
			RADN: 31	47.5 ± 9.34	15/16	24.0 ± 2.11	3/28	6/31	
Windisch et al. (2022) [13]	Retrospective	Switzerland	LDN: 104	54.1 ± 11	34/70	25.2 ± 4	50/54	17/104	NA
			RADN: 72	51.3 ± 11	22/50	24.9 ± 3	13/59	12/72	

### Risk of bias assessment for included studies

The included articles were published between 2002 and 2022. Eleven studies were retrospective, five were randomized-controlled trials (RCTs), and one was prospective. Of the 12 non-randomized studies, five had serious bias, four had moderate bias, and three had a low risk of bias. Of the five RCTs, one had low overall bias and four had some concerns of bias (Supplementary Table 3). The subgroup analysis included six articles, which were published between 2015 and 2022. Of these six studies, four were retrospective and two were RCTs. The two RCTs had some concerns of bias and the four retrospective studies had low to moderate risk of bias (Supplementary Table 3). The quality of evidence for every outcome was rated as extremely low according to GRADE (Supplementary Tables 4 and 5). Publication bias for each outcome was assessed using the Egger's and Peters tests with funnel plots (Supplementary Table 6 and Supplementary Figs. 2–10).

### Outcomes of interests

#### Estimated blood loss

Estimated blood loss was reported in 1,792 donors from five studies – 1,559 in the LDN group and 233 in the RADN group. A random effects model revealed that estimated blood loss was significantly lower in the LDN group (66.08 ml) than in the RADN group (95.43 ml) *(p* < 0.01, MD: – 13.28, 95% CI: [– 17.36, – 9.19], Supplementary Fig. 11). The heterogeneity of the pooled studies was not significant (I^2^ = 0%, P = 0.77). Estimated blood loss was not compared between experienced and inexperienced surgeons in these studies.

#### Conversion to open surgery

**Fig. 1:**
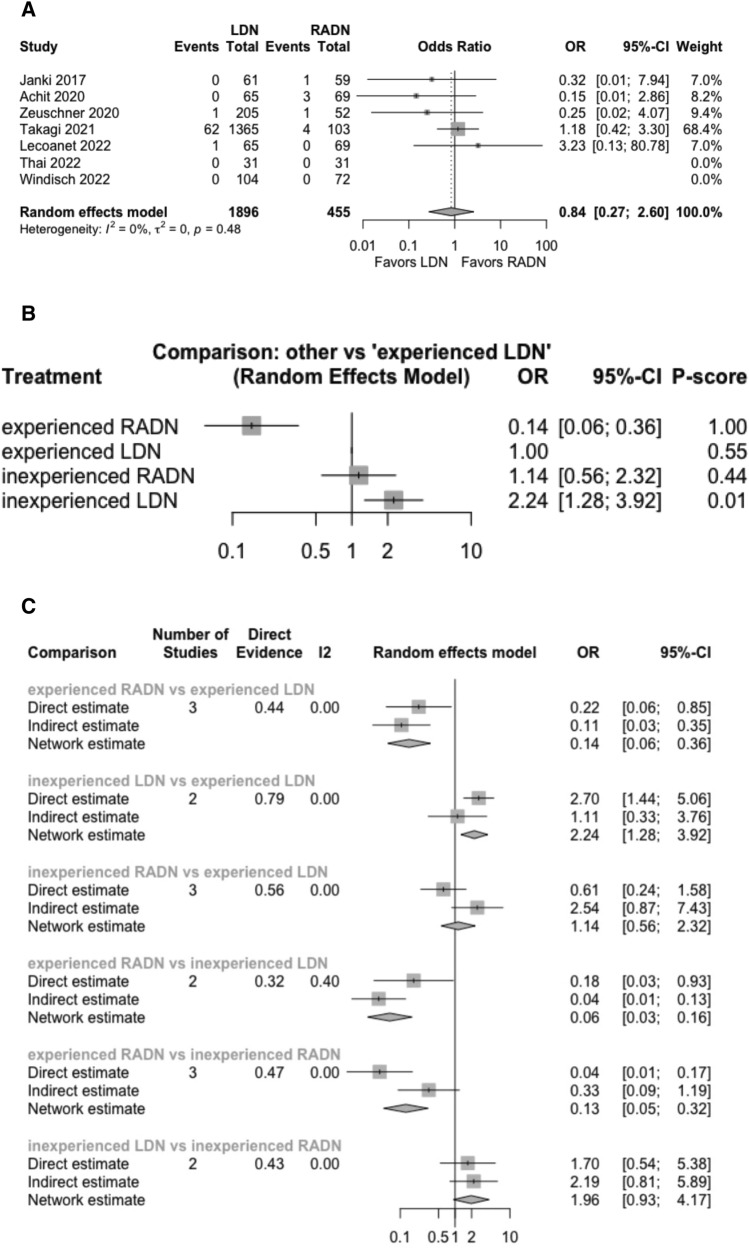
**A** Forest plot comparing conversion rate to open surgery between LDN and RADN donor groups using a Mantel–Haenszel random-effects model for meta-analysis. Odds ratios are presented with 95% confidence intervals. **B, C** Subgroup analysis comparing rate of conversion to open surgery between four subgroups based on surgical experience using a random-effects model for frequentist network meta-analysis. Odds ratios are presented with 95% confidence intervals

Seven studies reported the rate of conversion to open surgery in 2,351 donors – 1,896 in the LDN group and 455 in the RADN group. Conversion to open surgery occurred in 64/1,896 donors in LDN group (3.3%) and in 9/455 donors in RADN group (1.9%). The random effects model revealed no statistically significant difference in conversion to open surgery between the RADN and LDN groups *(p* > 0.05, OR: 0.84, 95% CI: [0.27, 2.6], Fig. 1A). The pooled studies were homogeneous *(*I^2^ = 0%, P = 0.48).

In the subgroup analysis, two studies reported the effect of the learning curve on conversion to open surgery. A random effects model network meta-analysis showed that conversion to open surgery was significantly lower in experienced RADN surgeons and experienced LDN surgeons than in inexperienced RADN surgeons and inexperienced LDN surgeons (*p* < 0.0001 and *p* < 0.01, respectively). In addition, conversion to open surgery was significantly lower in experienced RADN surgeons than in experienced LDN surgeons (OR: 0.1446, 95% CI: [0.0589; 0.3554], *p* < 0.0001, Fig. 1C). The network ranking test showed that experienced RADN surgeons were ranked first, followed by experienced LDN surgeons and inexperienced RADN surgeons (Fig. 1B).

#### Operation time

**Fig. 2:**
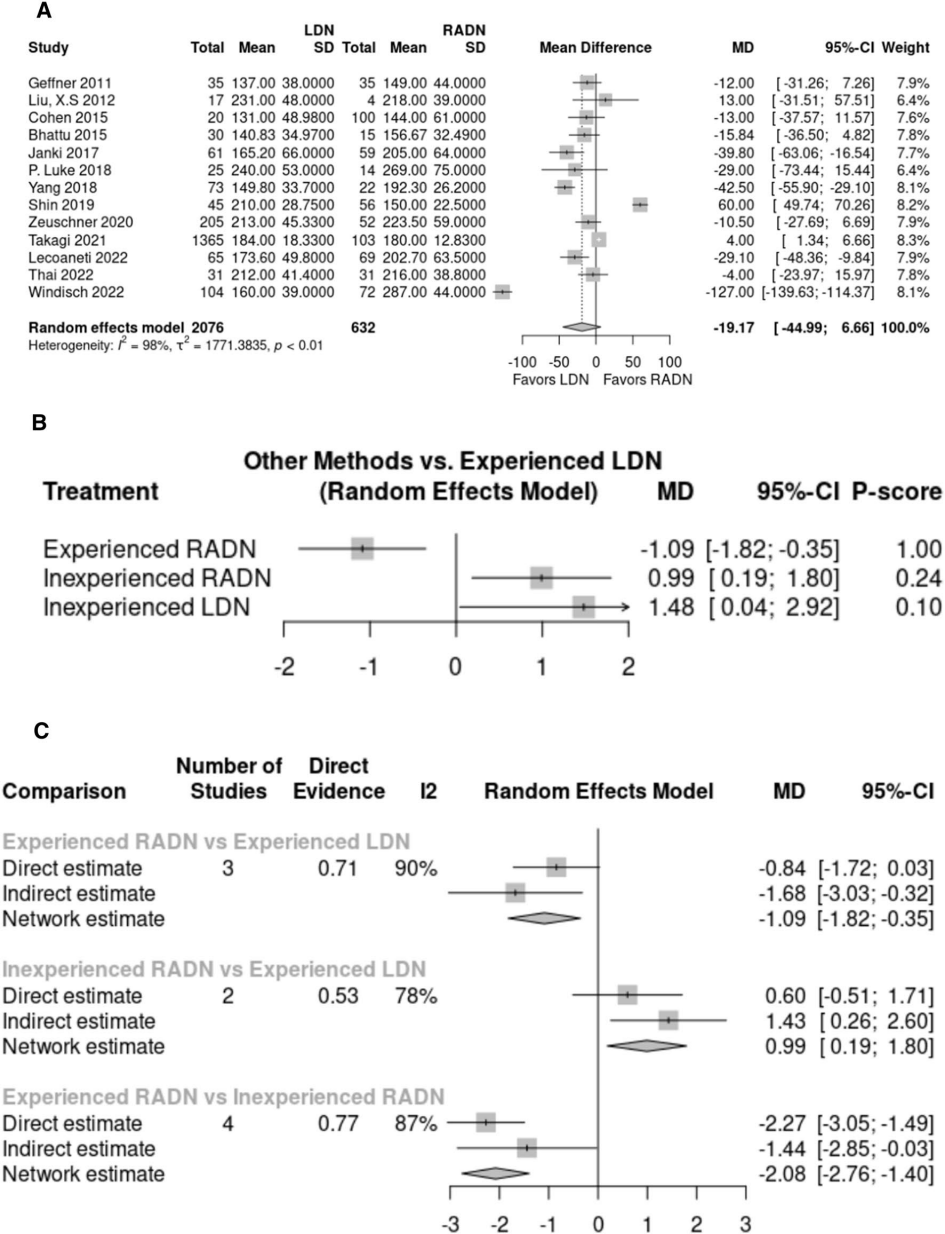
**A** Forest plot comparing operation time between LDN and RADN donor groups using a random-effects model for meta-analysis. Mean differences are presented with 95% confidence intervals. **B, C** Subgroup analysis comparing operation time between four subgroups based on surgeon’s experience using a random-effects model for frequentist network meta-analysis. Mean differences are presented with 95% confidence intervals

Operation time was reported in 13 studies with 2,708 donors – 2,076 in the LDN group and 632 in the RADN group. A random effects model showed no statistically significant difference in operation time between the RADN group (194.36 min) and the LDN group (183.69 min) (*p* > 0.05, MD: – 19.17, 95% CI: [– 44.99, 6.66], Fig. 2A). Pooled studies were heterogeneous (I^2^ = 98%, P < 0.01).

The effect of the learning curve on operation time was evaluated in all six studies included in our multi-arm subgroup analysis. Random effects model network meta-analysis showed that the operation time was significantly lower in the experienced RADN group than in the experienced LDN group (MD: – 1.09, 95% Cl: [– 1.82; – 0.35], *p* < 0.01) and the inexperienced RADN group (MD: 0.99, 95% Cl: [0.19; 1.80], *p* < 0.01). Based on *p*-values after the network ranking test, experienced RADN surgeons were ranked first, followed by experienced LDN surgeons, inexperienced RADN surgeons, and inexperienced LDN surgeons (Fig. 2B, C).

#### Overall surgical complications

**Fig. 3:**
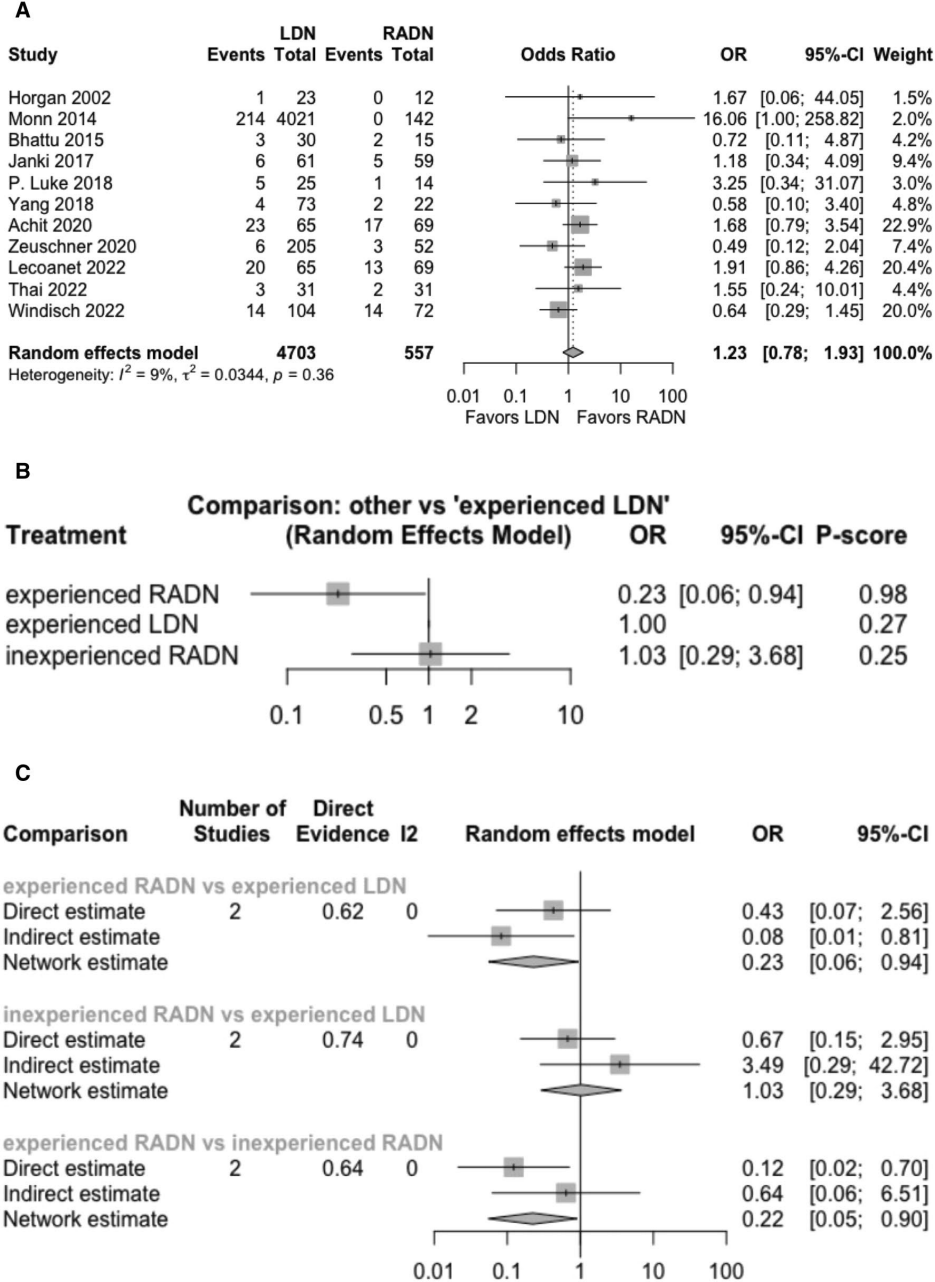
**A** Forest plot comparing overall surgical complications between LDN and RADN donor groups using a Mantel–Haenszel random-effects model for meta-analysis. Odds ratios are presented with 95% confidence intervals. **B, C** Subgroup analysis comparing overall surgical complications between four subgroups based on surgical experience using a random-effects model for frequentist network meta-analysis. Odds ratios are presented with 95% confidence intervals

Overall surgical complications were reported in 11 studies with 5,260 donors – 4,703 in the LDN group and 557 in the RADN group. These complications included intraoperative and postoperative complications and were experienced by 299/4,703 donors in the LDN group (6.3%) and 59/557 donors in the RADN group (10.5%). Random effects model analysis showed no differences in overall surgical complications between the RADN group and the LDN group (*p* > 0.05, OR: 1.23, 95% CI: [0.78, 1.93], Fig. 3A). Data from the eight pooled studies were homogeneous (I^2^ = 9%, P = 0.36).

Subgroup analysis was carried out to clarify the effect of the learning curve on overall surgical complications in RADN and LDN groups. Two of the four studies that provided data on overall surgical complications were included in this subgroup analysis. Our random-effects model network meta-analysis revealed significantly fewer overall surgical complications in the experienced RADN group than in the experienced LDN group and inexperienced RADN group (*p* < 0.05). There was no statistically significant difference in overall surgical complications between the experienced LDN group and inexperienced LDN group (*p* > 0.05). These findings are summarized in Fig. 3B, C. The experienced RADN group was ranked first followed by the experienced LDN group and inexperienced RADN group (Fig. 3B).

#### Major surgical complications

Major surgical complications were reported in five studies with 2,058 living donors – 1,778 in the LDN group and 280 in the RADN group. Major surgical complications occurred in 29/1,778 donors in the LDN group (1.6%) and in 6/280 donors in the RADN group (2%). The random-effects model revealed no significant differences in major surgical complications between the LDN group and the RADN group (*p* > 0.05, OR: 0.73, 95% CI: [0.25, 2.14], Supplementary Fig. 12A). Data from the three pooled studies were homogeneous (I^2^ = 0%, P = 0.69). Two studies reporting major surgical complications were included in subgroup analysis. The random effects network meta-analysis showed no significant difference in major complications between experienced and inexperienced RADN and LDN surgeons (*p* > 0.05, Supplementary Figs. 12B and 12C).

#### Length of hospital stay

**Fig. 4:**
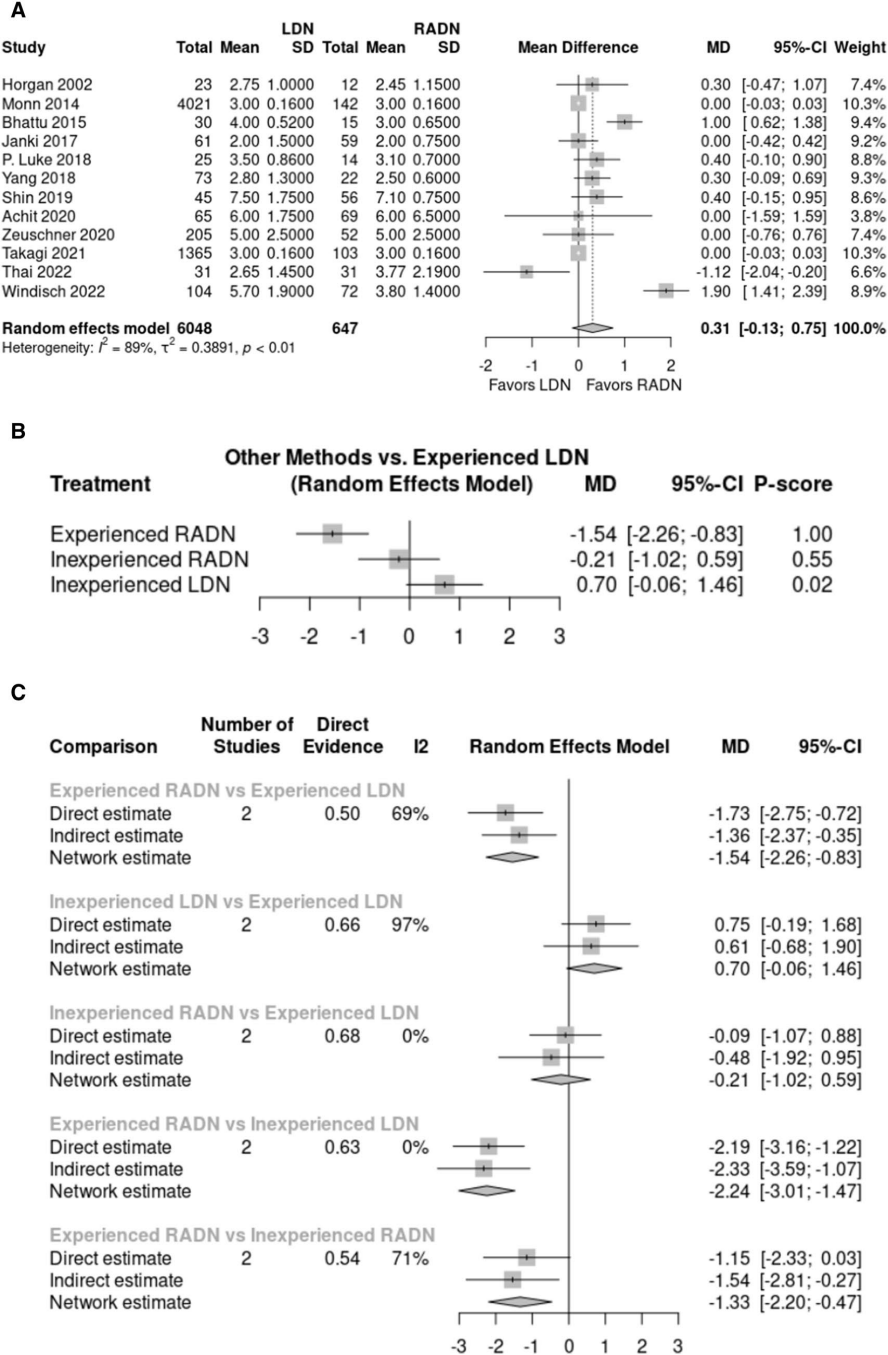
**A** Forest plot comparing length of hospital stay between LDN and RADN donor groups using a random-effects model for meta-analysis. Mean differences are presented with 95% confidence intervals. **B, C** Subgroup analysis comparing length of hospital stay between four subgroups based on surgical experience using a random-effects model for frequentist network meta-analysis. Mean differences are presented with 95% confidence intervals

The length of hospital stay was reported in 12 studies with 6,695 donors – 6,048 in the LDN group and 647 in the RADN group. The length of hospital stay was not significantly different between the RADN group (2.96 days) and the LDN group (2.99 days) (*p* > 0.05, MD: 0.31, 95% CI: [– 0.13, 0.75], Fig. 4A). High heterogeneity was shown after meta-analysis (I^2^ = 89%, P < 0.01). The effect of surgical experience on the length of hospital stay in the RADN and LDN groups was reported in two studies. Subgroup analysis revealed that more surgical experience of surgeon attenuated hospital stay in the RADN group (*p* < 0.05). Moreover, the hospital stay was significantly shorter in the experienced RADN group than in the experienced LDN group (MD: – 1.54, 95% CI: [– 2.26; – 0.83], *p* < 0.05). The results of this subgroup analysis are presented in Fig. 4B, C. Experienced RADN surgeons were ranked first followed by inexperienced RADN surgeons and experienced LDN surgeons (Fig. 4B).

#### Healthcare costs

Healthcare costs of RADN and LDN were compared in two studies with 4297 donors – 4086 in the LDN group and 211 in the RADN group. Our meta-analysis found no significant difference in healthcare costs between the RADN group ($34,000) and the LDN group ($36,500) (*p* > 0.05, MD: – 6.38, 95% CI: [– 72.90, 60.13], Supplementary Fig. 13). These results were heterogeneous (I^2^ = 100%, P = 0).

#### Warm ischemia time

**Fig. 5:**
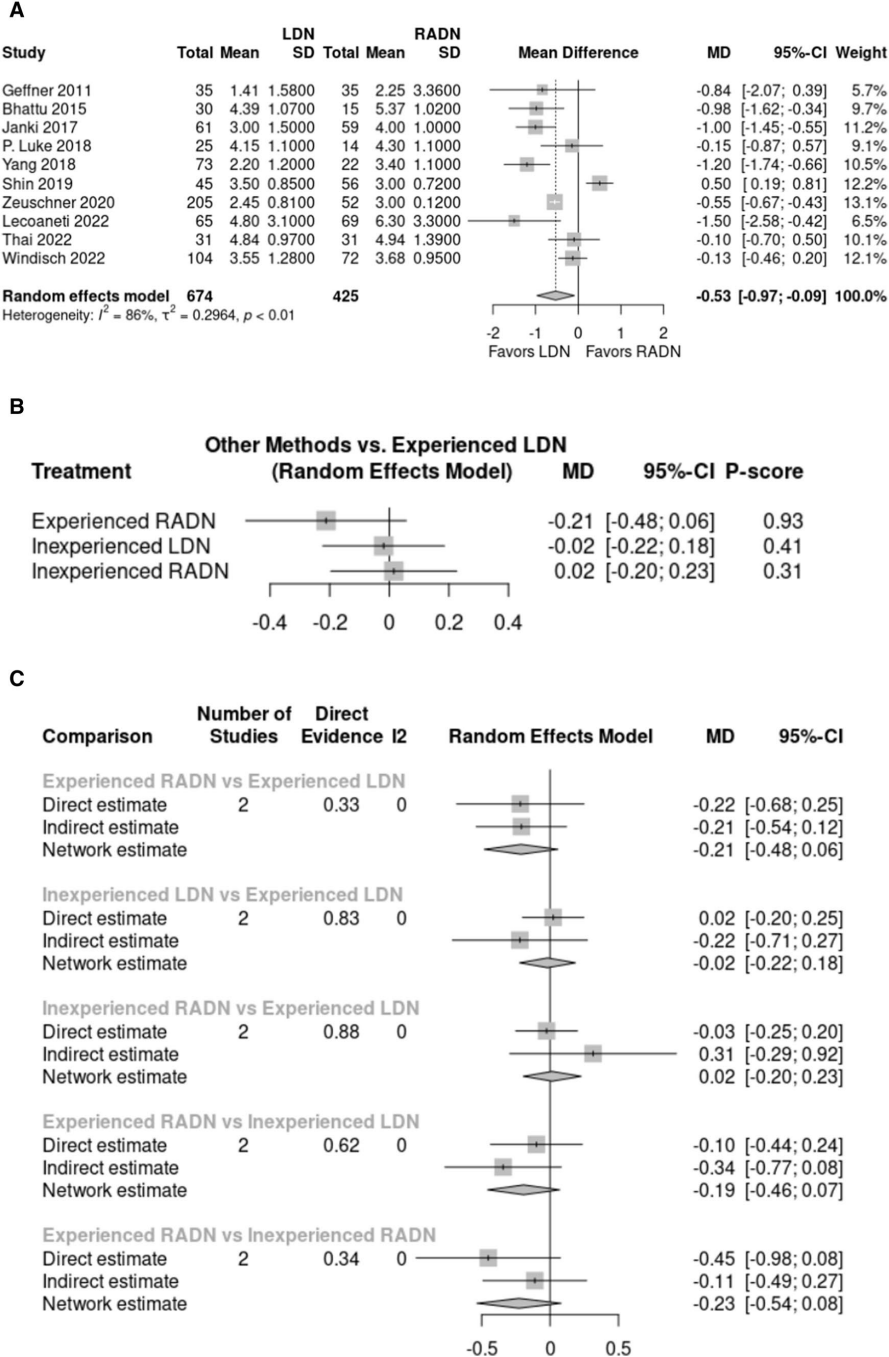
**A** Forest plot comparing warm ischemia time between LDN and RADN donor groups using a random-effects model for meta-analysis. Mean differences are presented with 95% confidence intervals. **B, C** Subgroup analysis comparing warm ischemia time between four subgroups based on surgical experience using a random-effects model for frequentist network meta-analysis. Mean differences are presented with 95% confidence intervals

The warm ischemia time was evaluated in 10 studies with 1,098 donors – 673 in the LDN group and 425 in the RADN group. Warm ischemia time was significantly shorter in the LDN group (3.14 min) than in the RADN group (4.01 min) (*p* < 0.05, MD: – 0.53, 95% CI: [– 0.97, – 0.09], Fig. 5A). Data from the ten pooled studies were heterogeneous (I^2^ = 86%, P < 0.01). Three studies reported on the influence of the learning curve on warm ischemia time in the LDN and RADN groups. Subgroups analysis revealed no significant difference in warm ischemia time between the groups (*p* > 0.05, Fig. 5B, C).

#### Delayed graft function

Delayed graft function was reported in five studies with 478 recipients. The random effects model revealed no differences in delayed graft function between the LDN and RADN groups (*p* > 0.05, Supplementary Fig. 14). Pooled studies were homogeneous (I^2^ = 0%, P = 0.54). No studies examined the effect of surgical experience on delayed graft function.

## Discussion

Minimally invasive techniques have long been recognized as the method of choice for donor nephrectomy [17]. The increasing use of robots in urologic and general surgeries has led to a number of systematic reviews and meta-analyses comparing the outcomes of RADN and LDN [5, 8]. These studies have shown that surgical experience directly affects the outcomes of kidney transplantation [18]. However, the effect of surgical experience on outcomes have not been compared between LDN and RADN. In the current meta-analysis, we assessed the role of surgical experience on outcomes following LDN and RADN in living donors to determine the effect of the learning curve on transplantation outcomes. We found no differences in surgical outcomes between the RADN and LDN groups. However, once the learning curve was completed, RADN was associated with better perioperative results than LDN was, including shorter operation time, lower rates of conversion to open surgery, fewer surgical complications, and shorter hospital stay. These findings indicate that RADN should be the method of choice for minimally invasive donor nephrectomy once the learning curve is complete.

Kidney transplantation from a living donor has been associated with better short- and long-term outcomes than cadaveric kidney transplantation. However, the health and safety of donors is an important issue [19]. Different minimally invasive methods have been used to extract kidneys from living donors, including LDN and RADN. A disadvantage of LDN is the lack of maneuverability and mobility during surgery [20] as well as the longer warm ischemia and operation time compared with open donor nephrectomy [21]. The shorter warm ischemia time offered by open donor nephrectomy could make this a better technique for pediatric kidney transplantation, where long-term graft survival is important, although this is still a matter of debate [22–24]. LDN is better than open donor nephrectomy for donors because it is associated with a faster return to normal physical activity [25].

To improve the problems with surgical mobility associated with LDN, RADN has been introduced as an alternative technique [26]. However, studies disagree on which of these two techniques is best; some have reported that RADN leads to poorer outcomes, such as prolonged warm ischemia time, longer operation time, more blood loss, and a higher rate of surgical complications and delayed graft function [12]. In the current study, we hypothesized that these poorer outcomes may be due to a lack of surgical experience, and that differences in surgical experience between studies may explain the conflicting findings. Interestingly, we found that surgical outcomes of RADN improved significantly once surgeons had gained sufficient experience. We found no differences in healthcare costs and delayed graft function between the RADN and LDN groups. Although the costs of surgical equipment are expected to be greater in the RADN group, this may be offset by the shorter hospital stay in these patients. These findings suggest that RADN may improve outcomes when performed by surgeons with sufficient experience without increasing healthcare costs. Similar findings have been reported from ROLARR trial in robot-assisted versus laparoscopic rectal surgery after adjusting for the learning effect. Although the primary outcomes of this trial could not show a significant difference between robot-assisted and laparoscopic surgery regarding conversion to open surgery, the adjusted analysis revealed a significant lower rate of conversion in robotic rectal surgeries performed by experienced surgeons [27].

Our results showed that the warm ischemia time was shorter in the LDN group than in the RADN group, but this difference disappeared once the surgeons had gained sufficient experience. These findings are in agreement with those of previous studies showing a shorter warm ischemia time for LDN than for RADN. Bhattu et al. suggested that the longer warm ischemia time for RADN could be explained by the undocking of the fourth arm during retrieval [28]. The operation time and warm ischemia time may also be prolonged in RADN because the primary attending surgeon operates the robot while a second attending surgeon extracts the kidney [8]. Our findings show that these problems can be solved by surgical experience and completion of the learning curve.

We observed no differences in operation time between the LDN and RADN groups, but our subgroup analysis showed that the operation time was significantly shorter in the experienced RADN group than in the experienced LDN group. Another meta-analysis published in 2018 reported a shorter operation time in the LDN group than the RADN group. Our findings suggest that this difference may be explained by differences in the level of surgical experience between the groups [29]. In support of our findings, a French RCT showed that operation time decreases with increasing surgical experience in RADN [11]. Similarly, another study found that the operation time for RADN decreased as the operating team became more familiar with the equipment and process of robotic surgery [30].

Our results showed lower blood loss in donors who underwent LDN than in donors who underwent RADN. However, this difference of blood loss between LDN group (66 ml) and RADN group (99 ml) was not clinically significant. A lack of surgical experience in the RADN may explain the higher blood loss, but there was insufficient data to confirm this in our subgroup analysis.

In agreement with a previous meta-analysis, we observed no differences in the rate of conversion to open surgery between the two groups [8]. However, when the surgery was performed by experienced surgeons, the conversion rate to open surgery was significantly lower in the RADN group than in the LDN group. This suggests that the learning curve is an important factor in avoiding conversion to open surgery.

We observed no differences in overall surgical complications between the RADN and LDN groups. However, subgroup analysis showed a significantly lower rate of overall surgical complications following RADN than following LDN when the surgery was performed by experienced surgeons. This could be explained by less pressure and tension at port sites during RADN, reducing the stress placed on the abdominal wall [28]. Similar to earlier investigations, our analysis also showed a shorter length of hospital stay in the RADN group then in the LDN group when surgery was performed by experienced surgeons. This may also explain the lower rate of surgical complications in this group [13, 28].

There are some limitations to this study. First, we could not assess the effect of surgical experience on all surgical outcomes in our subgroup analysis due to insufficient data. Second, the definitions of experienced and inexperienced surgeons were different between the included studies. Another limitation of this analysis could be the small number of studies included in subgroup-analysis to evaluate the possible effect of surgeon’s experience on the comparison of surgical outcomes between RADN with LDN groups.

## Conclusions

In conclusion, our meta-analysis has shown that surgical experience could improve the intraoperative and postoperative outcomes of RADN more than those of LDN. Based on these findings, RADN could be considered the method of choice for living donor nephrectomy once surgeons have gained sufficient experience in robotic surgery.

## Supplementary Information

Below is the link to the electronic supplementary material.

Supplementary file1 (PDF 1050 KB)

## Data Availability

The data sets analyzed during the current study are available from the corresponding author upon a reasonable request.
