# Supplementary material for: Robot-assisted versus laparoscopic living donor nephrectomy: superior outcomes after completion of the learning curve
Source: J Robot Surg. 2023 Aug 2;17(5):2513–26. doi: 10.1007/s11701-023-01681-0 (PMC10492879; doi:10.1007/s11701-023-01681-0)
Supplement: Supplementary file 1 — Supplementary file1 (PDF 1050 KB) [file 11701_2023_1681_MOESM1_ESM.pdf]

Supplementary Material

Robot-assisted versus laparoscopic living donor nephrectomy: Superior outcomes after completion of the learning curve

Elias Khajeh[1], Rajan Nikbakhsh[1], Ali Ramouz[1], Ali Majlesara[1], Mohammad Golriz[1], Beat P. Müller-Stich[1], Felix Nickel[1], Christian Morath[2], Martin Zeier[2], Arianeb Mehrabi[1]

[1] Department of General, Visceral and Transplant Surgery, University Hospital Heidelberg, Germany.

[2] Department of Nephrology, University of Heidelberg, Heidelberg, Germany

Correspondence: Professor Dr. Arianeb Mehrabi FICS, FEBS, FACS
Head of the Division for Abdominal Transplantation
Department of General, Visceral and Transplant Surgery
University Hospital Heidelberg
Im Neuenheimer Feld 420
69120 Heidelberg, Germany
Tel.: +49 6221 56 6205;
Fax: +49 6221 56 33934;
E-mail: arianeb.mehrabi@med.uni-heidelberg.de
ORCID-ID: 0000-0003-2843-1390

Publication bias and sensitivity analysis

The funnel plot was used to investigate publication bias (Supplementary Figures 2–10). Statistical analysis of publication bias for the different outcomes was performed using Peters [1] and Egger's regression [2] tests. These regression tests revealed an asymmetry in overall surgical complications (p = 0.01, t-value = -3.01, Supplementary Table 6) and major surgical complications (p = 0.43, t-value = 0.67, Supplementary Table 6) among studies. To remove highly heterogeneous findings extracted from asymmetric studies from the analysis, Trim and Fill analysis was performed to clarify the stability of outcomes. These analyses revealed no significant changes in the heterogeneity of overall surgical complications and major surgical complications, showing that the results of these outcomes were stable (Supplementary Table 6). According to funnel plots and statistical test (Peters and Egger's regression tests), the other surgical outcomes were distributed symmetrically among studies, indicating that there was no publication bias in this meta-analysis (Supplementary Table 6).

Figure legends

Supplementary Figure 1. PRISMA flow chart showing selection of articles for review.

Supplementary Figure 2. Funnel plot of publication bias test: Estimated blood loss in donors.

Supplementary Figure 3. Funnel plot of publication bias test: Conversion to open surgery in donors.

Supplementary Figure 4. Funnel plot of publication bias test: Operation time in donors.

Supplementary Figure 5. Funnel plot of publication bias test: Overall surgical complications in donors.

Supplementary Figure 6. Funnel plot of publication bias test: Major surgical complications in donors.

Supplementary Figure 7. Funnel plot of publication bias test: Length of hospital stay in donors.

Supplementary Figure 8. Funnel plot of publication bias test: Healthcare costs in donors.

Supplementary Figure 9. Funnel plot of publication bias test: Warm ischemia time in donors.

Supplementary Figure 10. Funnel plot of publication bias test: Delayed graft function in recipients.

Supplementary Figure 11. Forest plot comparing estimated blood loss between LDN and RADN donor groups using a random-effects model for meta-analysis. Mean differences are presented with 95% confidence intervals.

Supplementary Figure 12. (A) Forest plot comparing major surgical complications between LDN and RADN donor groups using a Mantel–Haenszel random-effects model for meta-analysis. Odds ratios are presented with 95% confidence intervals. (B and C) Subgroup analysis comparing major surgical complications between four subgroups based on surgical experience using a random-effects model for frequentist network meta-analysis. Odds ratios are presented with 95% confidence intervals.

Supplementary Figure 13. Forest plot comparing healthcare costs between LDN and RADN donor groups using a random-effects model for meta-analysis. Mean differences are presented with 95% confidence intervals.

Supplementary Figure 14. Forest plot comparing delayed graft function between LDN and RADN recipient groups using a Mantel–Haenszel random-effects model for meta-analysis. Odds ratios are presented with 95% confidence intervals.

Supplementary Table 1. Grades of recommendation, assessment, development, and evaluation **(**GRADE) categories for quality of evidence.

GRADE Quality of evidence	Interpretation
High	Further research is very unlikely to change our confidence in the estimate of effect.
Moderate	Further research is likely to have an impact on our confidence in the estimate of effect and may change the estimate.
Low	Further research is very likely to have an impact on our confidence in estimate of effect and is likely to change the estimate.
Very low	Any estimate of effect is very uncertain.

Supplementary Table 2. Study characteristics of included studies in subgroup analysis.

Study (year)	Comparison	Number of patients				Cut-off for surgical experience (number of donor nephrectomies)		Outcomes assessed in study
		Inexperienced LDN	Experienced LDN	Inexperienced RADN	Experienced RADN	LDN	RADN	
Cohen et al. (2015)	experienced LDN **vs** inexperienced RADN / experienced LDN **vs** experienced RADN / inexperienced RADN **vs** experienced RADN	NA	20	20	80	NA	20	Operation time, healthcare costs
Zeuschner et al. (2021)	inexperienced LDN **vs** experienced LDN **/** inexperienced RADN **vs** experienced RADN	26	26	102	103	102	26	Operation time, major surgical complications based on Clavien–Dindo grade ≥3, length of hospital stay, conversion to open surgery
Dumlu et al. (2021)	experienced LDN **vs** inexperienced RADN / experienced LDN **vs** experienced RADN / inexperienced RADN **vs** experienced RADN	NA	20	20	20	NA	20	Operation time, overall surgical complications, warm ischemia time, conversion to open surgery, laparoscopic system setup time
Takagi et al. (2021)	inexperienced LDN **vs** experienced LDN / inexperienced RADN **vs** experienced RADN	277	1088	46	57	23	26	Operation time, major surgical complications, length of hospital stay, conversion to open surgery
Lecoanet et al. (2022)	inexperienced RADN **vs** experienced RADN	NA	NA	28	41	NA	28	Operation time, warm ischemia time
Windisch et al. (2022)	inexperienced LDN **vs** inexperienced RADN / experienced LDN **vs** experienced RADN	46	58	24	48	46	24	Operation time, overall surgical complications, major surgical complications (Clavien–Dindo grade ≥3), length of hospital stay, warm ischemia time

Supplementary Table 3. Quality assessment of the included studies.

Study	Risk of bias							
	**Bias due to confounding**	**Bias in selection of participants into the study**	**Bias in classification of interventions**	**Bias due to deviations from intended interventions**	**Bias due to missing data**	**Bias in measurement of outcomes**	**Bias in selection of the reported result**	**ROBINS-I (overall)**
Horgan (2002)	Serious	Moderate	Low	Low	Moderate	Low	Moderate	Serious
Geffner (2011)	Low	Low	Low	Low	Low	Low	Low	Low
Liu, X.S (2012)	Serious	Low	Low	Low	Moderate	Low	Moderate	Serious
Monn (2014)	Serious	Moderate	Low	Low	Moderate	Serious	Moderate	Serious
Cohen (2015)	Moderate	Low	Low	Low	Moderate	Low	Moderate	Moderate
Janki (2017)	Low	Low	Low	Low	Low	Low	Low	Low
Yang (2018)	Moderate	Moderate	Low	Moderate	Moderate	Low	Moderate	Serious
Shin (2019)	Moderate	Moderate	Low	Low	Moderate	Low	Moderate	Moderate
Zeuschner (2021)	Low	Low	Low	Low	Low	Low	Low	Low
Takagi (2021)	Moderate	Low	Low	Low	Moderate	Low	Moderate	Moderate
Thai (2022)	Serious	Low	Low	Low	Moderate	Low	Moderate	Serious
Windisch (2022)	Moderate	Low	Moderate	Low	Moderate	Low	Moderate	Moderate
**Cochrane risk of bias tool for randomized controlled trials**								
**First author**	**Bhattu (2015)**	**P.Luke (2018)**	**Achit (2020)**	**Dumlu (2021)**	**Lecoanet (2022)**			
Bias arising from the randomization process	Low risk	Some concerns	Some concerns	Some concerns	Some concerns			
Bias arising from the timing of identification and recruitment of individual participants in relation to timing of randomization	Low risk	Some concerns	Low risk	Some concerns	Some concerns			
Bias due to deviations from intended interventions	Low risk	Some concerns	Low risk	Some concerns	Some concerns			
Bias due to missing outcome data	Low risk	Low risk	Low risk	Some concerns	Some concerns			
Bias in measurement of the outcome	Low risk	Some concerns	Some concerns	Some concerns	Some concerns			
Bias in selection of the reported result	Low risk	Some concerns	Some concerns	Some concerns	Some concerns			
Overall bias	Low	Some concerns	Some concerns	Some concerns	Some concerns			

Supplementary Table 4. Grades of recommendation, assessment, development, and evaluation **(**GRADE) assessment for outcomes of interest

Comparison	Studies	GRADE							
		**Risk of bias**	**Inconsistency**	**Indirectness**	**Imprecision**	**Publication bias**	**Large or moderate effect size**	**Exposure-response gradient**	**Overall quality**
Laparoscopic donor nephrectomy vs. robot-assisted donor nephrectomy									
Estimated blood loss	2	Serious	Serious	Not serious	Serious	Not serious	Not present	Not present	Very low
Conversion to open surgery	7	Serious	Not serious	Not serious	Serious	Not serious	Not present	Not present	Very low
Operation time	13	Serious	Serious	Not serious	Serious	Serious	Not present	Not present	Very low
Overall surgical complications	11	Very serious	Not serious	Not serious	Serious	Not serious	Not present	Not present	Very low
Major surgical complications	5	Serious	Not serious	Serious	Serious	Not serious	Not present	Not present	Very low
Length of hospital stay	12	Very serious	Serious	Not serious	Serious	Serious	Not present	Not present	Very low
Healthcare costs	2	Very serious	Serious	Not serious	Serious	Serious	Not present	Not present	Very low
Warm ischemia time	10	Serious	Serious	Not serious	Serious	Serious	Not present	Not present	Very low
Delayed graft function	5	Serious	Not serious	Not serious	Serious	Not serious	Not present	Not present	Very low

**Supplementary Table 5.** Meta-analysis results and quality of evidence for outcomes				
**Outcome**	**Number of studies**	**Pooled relative effect (95% CI)**	**Statistical heterogeneity (%); *p*-value**	**Quality of evidence (GRADE)**
Laparoscopic donor nephrectomy vs. robot-assisted donor nephrectomy				
Estimated blood loss	5	MD = -13.28 (-17.36; -9.19)	0%; $p = 0.77$	Very low
Conversion to open surgery	7	OR = 0.84 (0.27; 2.60)	0%; $p = 0.48$	Very low
Operation time	13	MD = -19.17 (44.99; 6.66)	98%; $p < 0.01$	Very low
Overall surgical complications	11	OR = 1.23 (0.78; 1.93)	9%; $p = 0.36$	Very low
Major surgical complications	5	OR = 0.73 (0.25; 2.14)	0%; $p = 0.69$	Very low
Length of hospital stay	12	MD = 0.31 (-0.13; 0.75)	89%; $p < 0.01$	Very low
Health care costs	2	MD = -6.38 (-72.90; 60.13)	100%; $p = 0$	Very low
Warm ischemia time	10	MD = -0.53 (-0.97; -0.09)	86%; $p < 0.01$	Very low
Delayed graft function	5	OR = 0.55 (0.11–2.69)	0%; $p = 0.54$	Very low
OR: Odd's ratio; MD: mean difference				

**Supplementary Table 6.** Evaluation of publication bias					
**Parameters**	**Peters test**		**Egger's regression test**		**Trim and fill analysis (in case of significant asymmetry)**
	p	**t-value**	***p***	**t-value**	
Estimated blood loss	NA*		0.27	1.36	
Conversion to open surgery	0.15	-1.87	0.33	-1.16	
Operation time	NA		0.58	-0.57	
Overall surgical complications	0.01	-3.01	0.67	0.43	OR = 1.23(0.78; 1.93), $p = 0.33$
Major surgical complications	0.26	-1.54	0.01	7.03	OR = 0.62 (0.19; 1.95), $p = 0.26$
Length of hospital stay	NA		0.23	1.28	
Healthcare costs	NA		NA**		
Warm ischemia time	NA		0.92	-0.10	
Delayed graft function	0.78	-0.35	0.86	0.21	

* Peters test has been only defined
** For outcomes with less than 3 studies involved, it is not possible to perform Egger's regression intercept test.

Supplementary Figure 1.

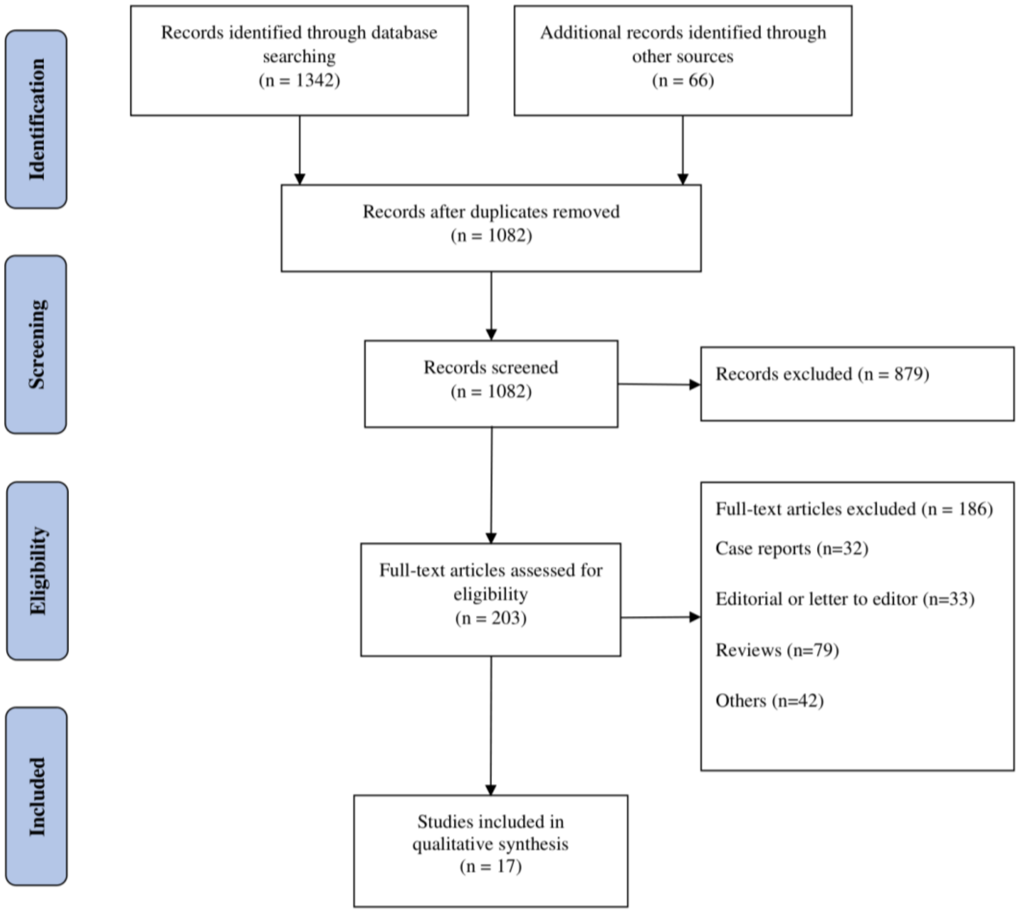

Supplementary Figure 2.

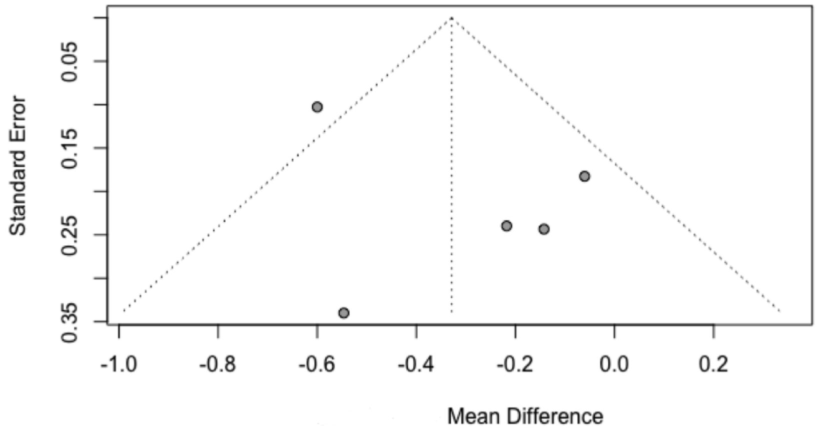

Supplementary Figure 3.

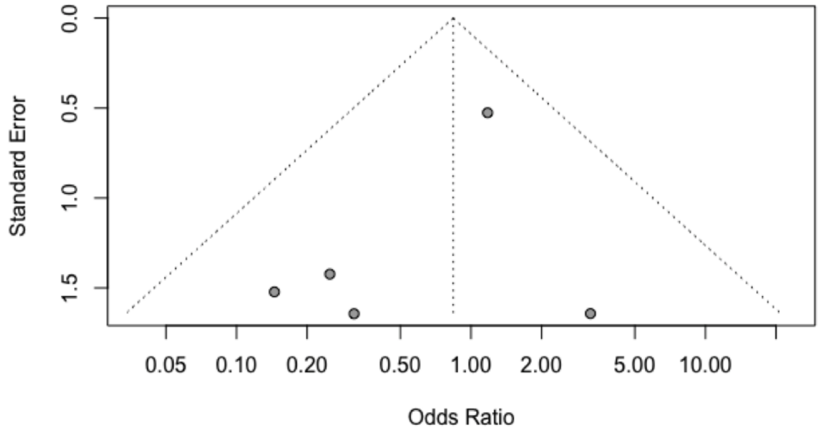

Supplementary Figure 4.

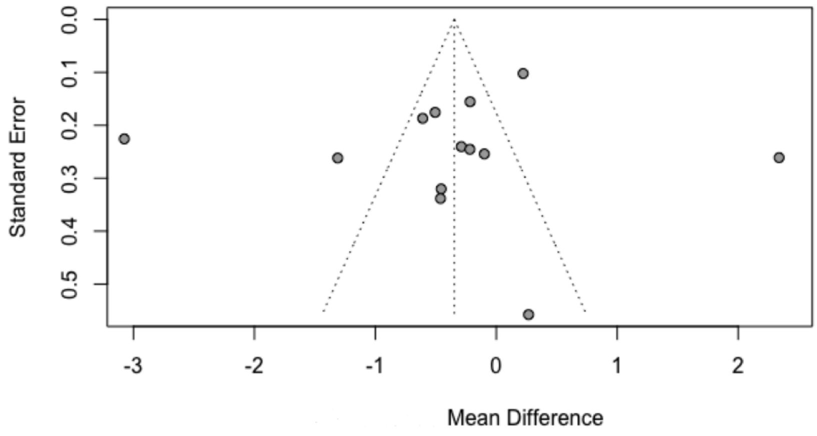

Supplementary Figure 5.

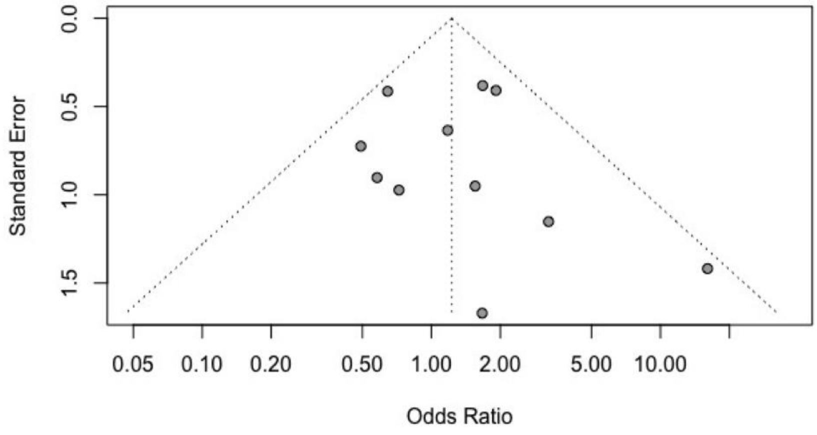

Supplementary Figure 6.

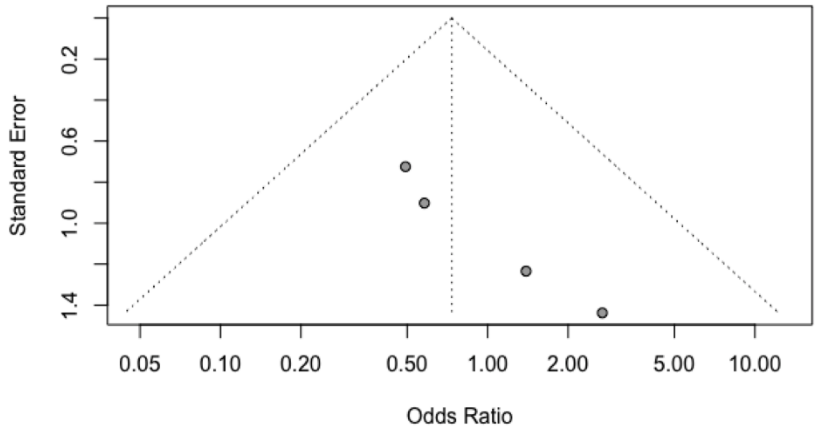

Supplementary Figure 7.

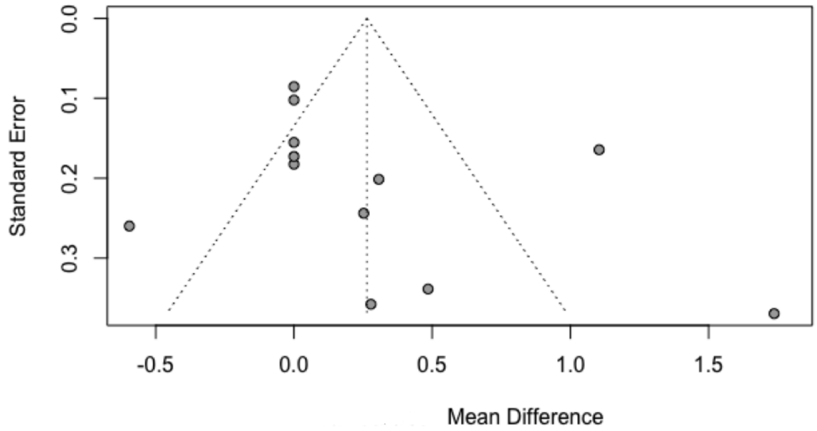

Supplementary Figure 8.

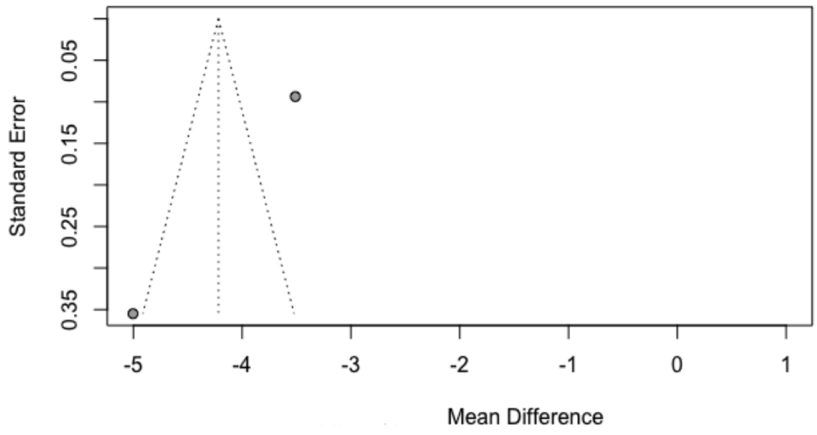

Supplementary Figure 9.

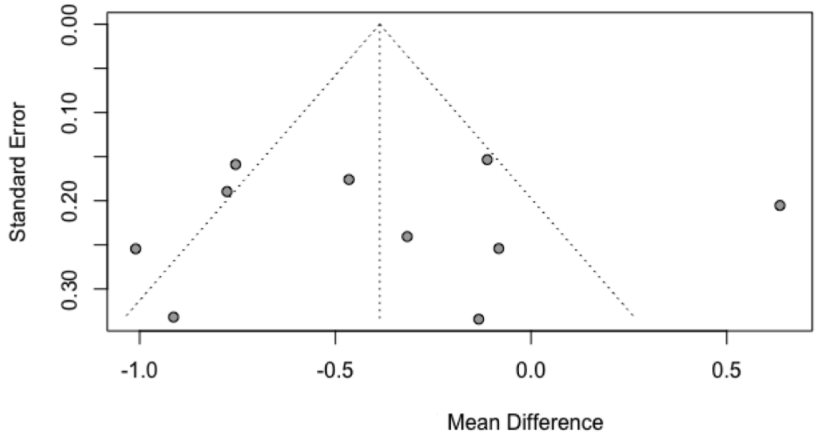

Supplementary Figure 10.

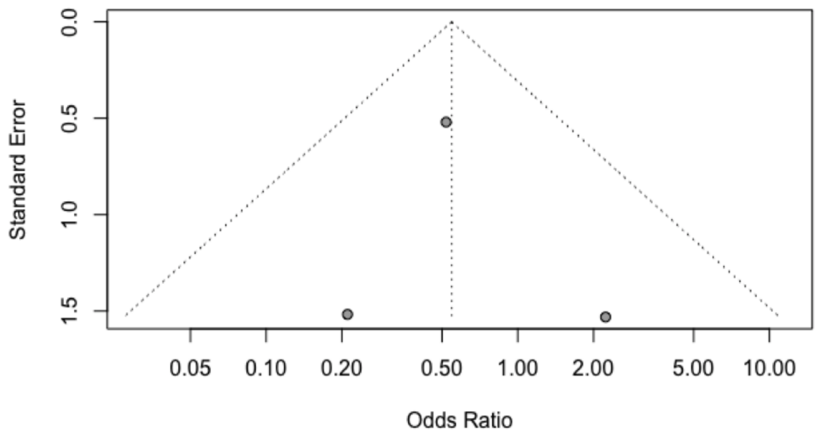

Supplementary Figure 11.

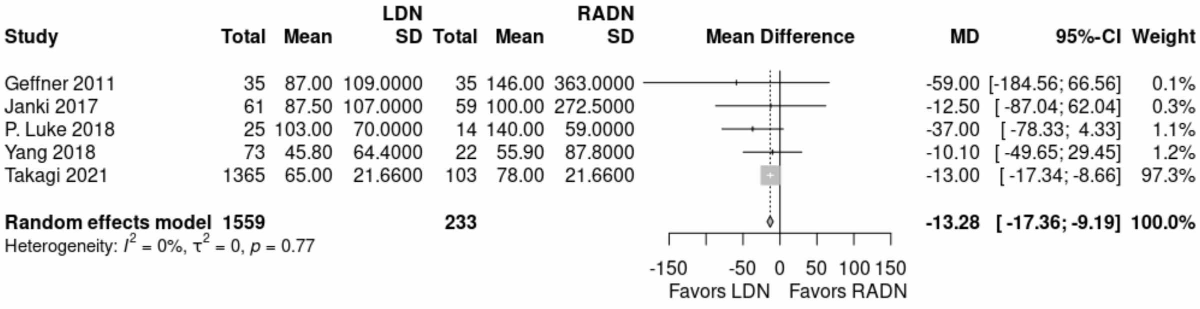

Supplementary Figure 12.

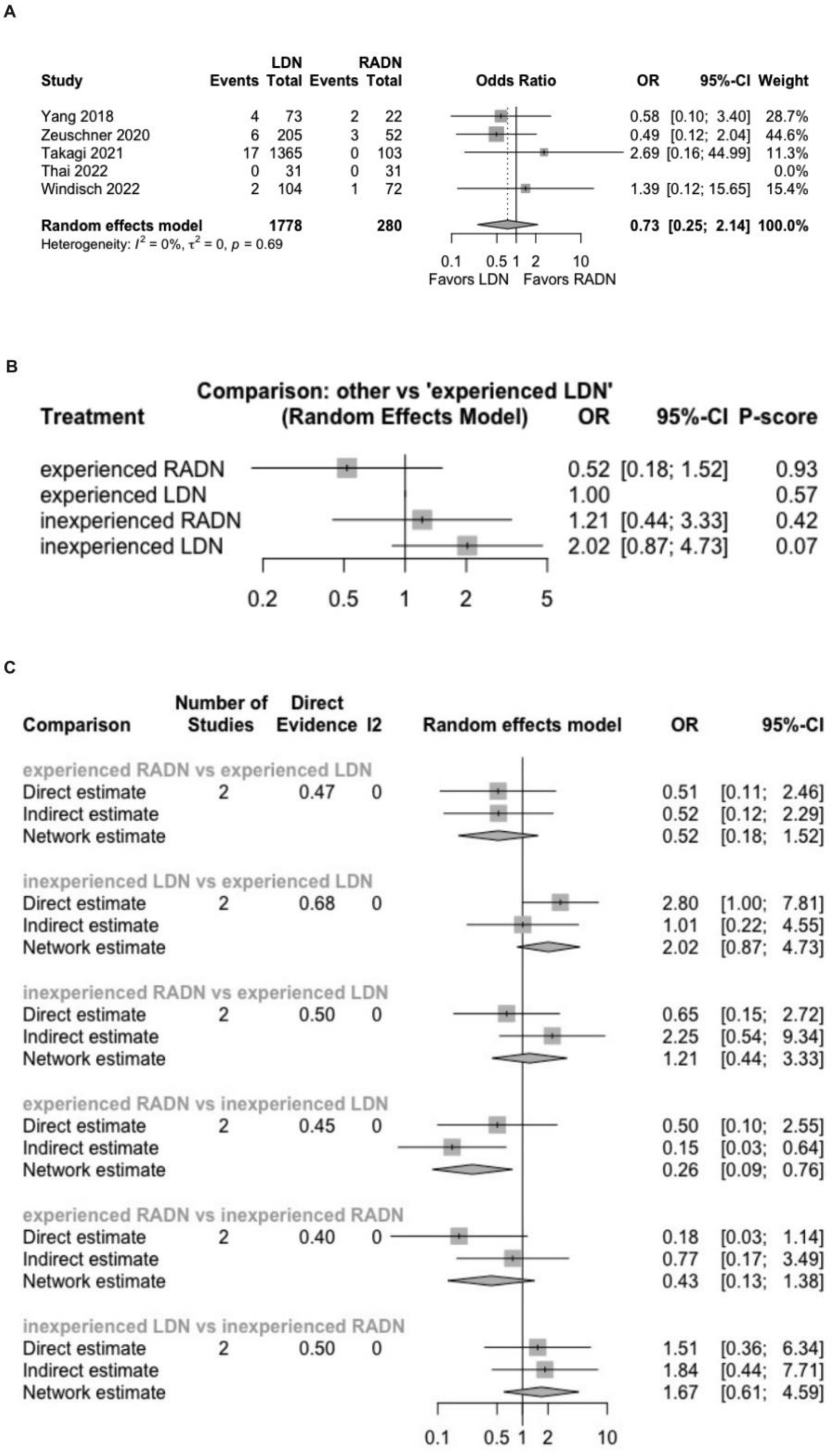

Supplementary Figure 13.

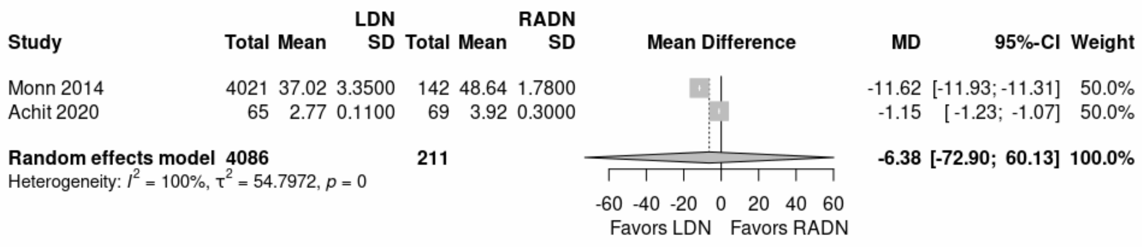

Supplementary Figure 14.

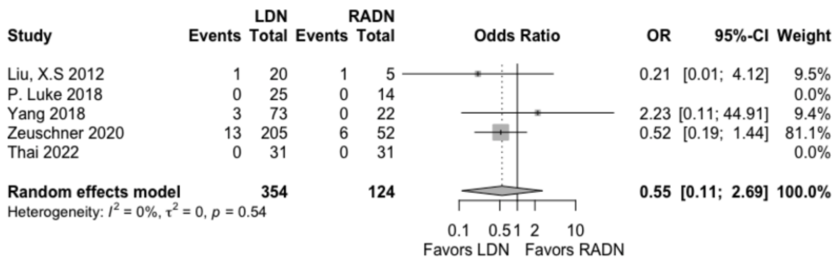

References

1. Peters JL, Sutton AJ, Jones DR, Abrams KR, Rushton LJSim. Performance of the trim and fill method in the presence of publication bias and between-study heterogeneity. 2007;26(25): 4544-4562.

2. Egger M, Smith GD, Schneider M, Minder CJB. Bias in meta-analysis detected by a simple, graphical test. 1997;315(7109): 629-634.
